# Optical Charge State Manipulation of Lead-Vacancy Centers in Diamond

**DOI:** 10.1021/acs.nanolett.5c04448

**Published:** 2025-11-13

**Authors:** Yiyang Chen, Yoshiyuki Miyamoto, Eiki Ota, Ryotaro Abe, Takashi Taniguchi, Shinobu Onoda, Mutsuko Hatano, Takayuki Iwasaki

**Affiliations:** † Department of Electrical and Electronic Engineering, School of Engineering, 13290 Institute of Science Tokyo, Meguro, 152-8552 Tokyo Japan; ‡ Advanced Power Electronics Research Center, National Institute of Advanced Industrial Science and Technology, Tsukuba, 305-8568 Ibaraki Japan; § Research Center for Materials Nanoarchitectonics, National Institute for Materials Science, Tsukuba, 305-0044 Ibaraki Japan; ∥ Takasaki Advanced Radiation Research Institute, National Institutes for Quantum Science and Technology, 1233 Watanuki, Takasaki, 370-1292 Gunma Japan

**Keywords:** Diamond, Lead-vacancy center, Charge state control, Quantum emitter, Quantum information

## Abstract

Group-IV vacancy centers in diamond exhibit excellent optical and spin coherence properties, making them highly promising and scalable spin qubit candidates. Since only specific charge states are magneto-optically active, control over the charge state is fundamental for quantum applications. Here, we realize the charge state control of lead-vacancy centers (PbV) through multicolor laser irradiation. We achieve tunable population manipulation of the negatively charged state from 0 to 89%, paving the way for spin control of the negatively charged PbV center. Furthermore, through analysis of charge state dynamics, we propose a charge cycle between the neutral and negatively charged states, indicating a possible pathway to the neutral PbV center with a spin-1 system.

## Graphical abstract

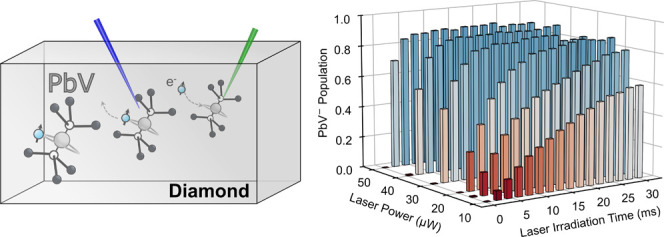

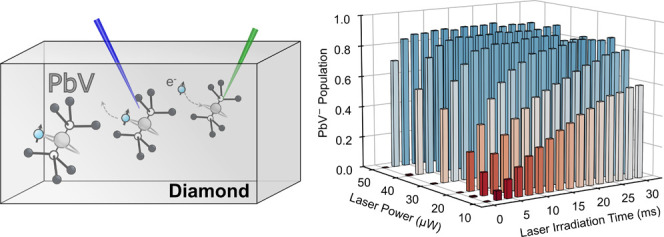

Quantum emitters in diamond have emerged as one of the leading platforms for quantum applications. Multinode quantum networks have been demonstrated based on nitrogen-vacancy (NV) centers.^1^ However, the fraction of zero-phonon line (ZPL) becomes as low as ∼3%, leading to a limited entanglement generation rate.^2^ To overcome this issue, the negatively charged group-IV color centers in diamond,^3–6^ including silicon-vacancy (SiV), germanium-vacancy (GeV), tin-vacancy (SnV), and lead-vacancy centers (PbV) with an inversion symmetry, have been proposed. These color centers exhibit higher Debye–Waller factors of 34–87%.^7–11^ Long distance entanglement has been generated between SiV centers,^12^ while they require cooling to the mK range for a long spin coherence time^13^ without a large strain.^14^ The negatively charged PbV center possesses the highest zero field splitting of ∼3900 GHz in the ground state, effectively suppressing the phonon interaction between the sublevels. Therefore, the transform-limited line width has been observed even at temperatures above 10 K.^11^ A spin coherence time on the millisecond level can be also expected at similar temperatures.^6^ Accordingly, the PbV center is an important building block for quantum network nodes. In addition to the negatively charged state, the neutral state of the group-IV color centers is an interesting quantum system owing to the spin-1 system, leading to a long spin coherence time at an even higher temperature, as demonstrated in the SiV center.^15^

Since only specific charge states are magneto-optically usable, understanding the charge transfer process and creating sufficient population in the desired charge states are fundamental for quantum application. Several approaches have been demonstrated for the charge state control of the color centers in diamond, including surface termination of diamond,^16–18^ doping with phosphorus^19^ or boron,^15^ electrical tuning in devices,^20–23^ photocarrier generation,^24–26^ and optical control.^27–32^ Surface termination can be only applicable to color centers close to the surface, and dopants in the lattice possibly degrade the optical properties,^15,19^ which significantly limits further practical applications. Among the methods above, optical irradiation is the simplest and highly controllable method, in which coherent emission can be expected.

For the PbV center, capture of photocarriers generated from optically excited defects have been shown to lead to the charge state transition under 532 nm irradiation.^33^ As the direct optical method, it has been known that 532 nm laser irradiation initializes PbV centers to the bright negatively charged state after their transition to a dark state under resonant excitation.^11^ However, the important information on the transition rate, population, and transition mechanism are totally missing, and it has not been revealed whether the charge state can be optically cycled with nonresonant visible lasers. In this work, we report the charge state cycle of PbV centers by multicolor nonresonant laser irradiation. We demonstrate that blue laser irradiation shelves the PbV centers into a pure dark state with a one-photon process, while the green laser irradiation repumps the dark state into the negatively charged state with a two-photon process. By combination of both lasers, we achieve directly and freely manipulating the charge state and population of the negatively charged state between around 0 to 89%, indicating that the green laser creates a sufficiently high population in the negatively charged state, toward high-fidelity spin control.^34^ Finally, a model of the charge dynamics is proposed with first-principles calculation.

Figure 1(a) shows the atomic structure of the PbV center. A lead impurity takes an interstitial position between carbon vacancies, possessing the inversion symmetry with a nearly zero first-order permanent electrical dipole moment.^35,36^ Under the combined influence of the spin–orbit interaction and Jahn–Teller effect,^37,38^ both ground and excited states of the negatively charged PbV center split into two sets of Kramers’ doublets (Figure 1 (b)). Consequently, four optical transition channels exist under zero magnetic field, mentioned as A-D. The C and D transitions have wavelengths of 550 and 554 nm, respectively, at a low temperature.^6^ The frequency difference between the C and D transitions gives a large zero-field splitting of approximately 3900 GHz in the ground state. The C transition shows a transform-limited line width of ∼39 MHz, while the D transition is significantly broadened due to the phonon interaction.^11^ Thus, we measure the C transition of PbV emitters under resonant excitation.

**Figure 1:**
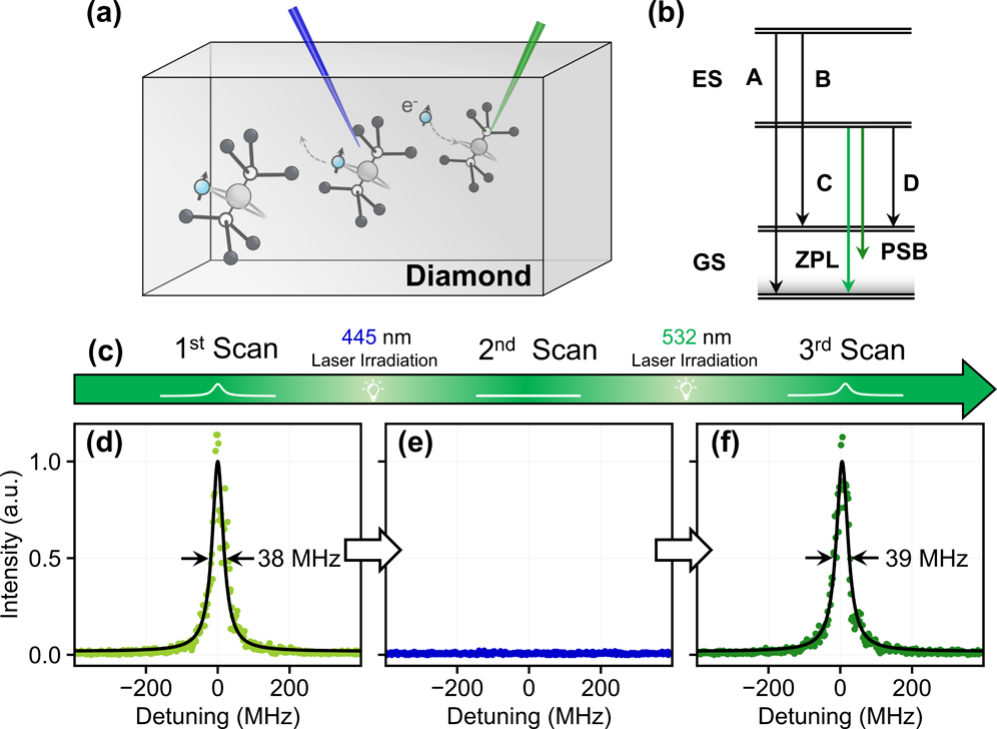
Charge state control of a PbV center using visible light laser irradiation. (a) PbV centers in diamond. A blue laser changes the charge state of the PbV^*‑*^ center to another dark state, while a green laser initializes to the −1 charged state. (b) Optical transitions of PbV^–^. GS and ES denote the ground state and excited state, respectively. (c) A sequence employed to investigate the influence of nonresonant lasers to the charge state of PbV. A total of three PLE scans are continuously performed, with 445 and 532 nm laser irradiation at off-resonant frequencies. (d) First PLE spectrum. (e) Second PLE spectrum after 445 nm laser irradiation. (f) Third PLE spectrum after 532 nm laser irradiation. The zero detuning of the three spectra is uniformly set to the central wavelength in the first scan in panel (d). The resonant laser power is set to 2 nW.

Two PbV samples (Sample I and II) are prepared by ion implantation and high-pressure and high-temperature (HPHT) annealing^33^ (see SI for experimental details). We investigate the charge state transition of a PbV center in Sample I under nonresonant laser irradiation (445 and 532 nm) using a sequence depicted in Figure 1(c). After the first photoluminescence excitation (PLE) is recorded with the resonant laser, a 445 nm laser (28.5 μW) is irradiated. Then, the second PLE is sequentially measured. Following another 532 nm laser irradiation (100 μW), we recorded the third PLE spectrum. To avoid simultaneous irradiation of the resonant and nonresonant lasers, which may cause additional charge state transition as observed in GeV and SnV centers,^30,31^ the nonresonant laser is irradiated when the detuning of the tunable laser exceeds at least 4 GHz. As shown in Figure 1(d), the first PLE spectrum shows a line width of 38 MHz by a Lorentzian function fitting, which agrees well with the transform-limited line width of PbV centers,^11^ indicating the high-quality formation of the PbV center. Note that in the following two PLE scans the zero detuning is uniformly set to the center wavelength of this first scan. In the second PLE scan, we do not see any peak in the scan range from +4.5 GHz to −7.2 GHz detuning. The measurement data around the zero detuning is shown in Figure 1(e). Finally, in the third scan after the application of the 532 nm laser irradiation, we again observe a resonant peak with a similar line width of 39 MHz at the photon frequency (Figure 1(f)). These observations indicate that the 445 nm laser irradiation shelves the negatively charged PbV center into a dark state through photoionization, leading to the disappearance of the PLE spectrum, while the 532 nm laser irradiation repumps the PbV center into the bright negatively charged state, resulting in the reappearance of the peak. Note that we do not observe the ZPLs of the PbV^–^ center under 445 nm irradiation in a PL spectrum (SI Figure S1), further manifesting the dark state transition of the PbV center.

To reveal the dynamics behind the charge state transitions induced by the nonresonant lasers, we conduct time-resolved pulse experiments. The sequence in Figure 2(a) is designed to investigate the charge state transition rate resulting from the 445 nm laser irradiation. Sixteen times repeated resonant and fifteen times repeated nonresonant pulses correspond to the readout of the PbV charge state and charge state control, respectively. When the PbV center possesses the negatively charged state, it can be resonantly excited, producing a substantial number of photons in the phonon sideband (PSB). On the other hand, the PbV center in the dark state cannot be resonantly excited, and thus, only background signals are detected. A subsequent 532 nm green pulse is initialized to the negatively charged state. In Figure 2(b), the fluorescence from the PbV^–^ gradually decays as the number of the 445 nm pulse increases. Finally, it goes down to the background level, corresponding to a dark state. It is well-known that resonant excitation also results in the transition to the dark state. However, we find that the resonant laser power and the excitation time used here for the charge state read-out have little impact on this phenomenon (SI Figure S2). The fluorescence is recovered with a 532 nm laser pulse, indicating the charge state initialization into a bright negatively charged state. Figure 2(c) shows measured fluorescence as a function of the total irradiation time of the 445 nm laser at various 445 nm laser powers. At all laser powers, the fluorescence decays as the irradiation time. The transition rates are obtained by fitting each curve with a monoexponential function. The power dependence of the transition rate is presented in Figure 2(d). A linear function fits well with a slope of 32 Hz/μW, indicating that the shelving process from the negatively charged state to the dark state is a one-photon process under 445 nm laser irradiation.

**Figure 2:**
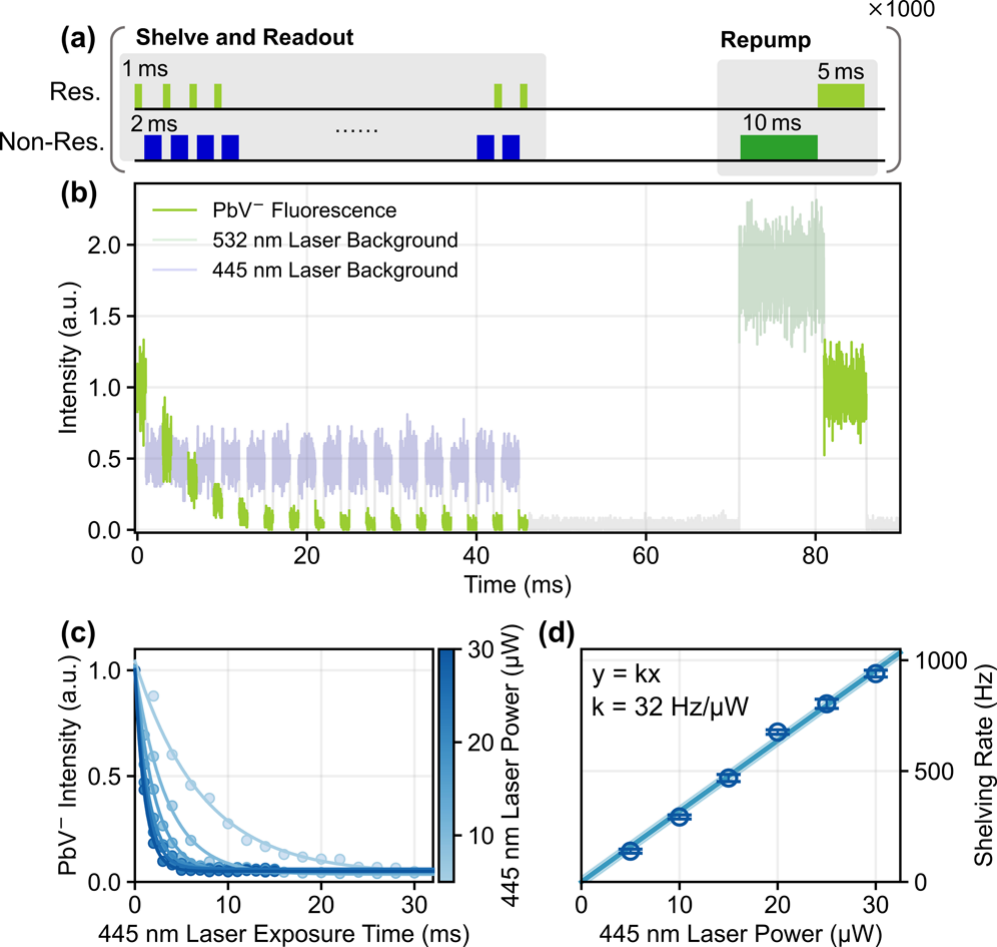
Time-resolved experiments of shelving process. (a) Pulse sequence. The 445 nm laser pulse width was set to 1 ms for high laser power. (b) Fluorescence from a negatively charged PbV center, controlled by 445 nm laser irradiation. (resonant laser: 2 nW, 445 nm laser: 10 μW, 532 nm laser: 100 μW) (c) Transition curves using different 445 nm laser powers. (d) Transition rate as a function of the 445 nm laser power. Error bars are from the fitting error in panel (c), and the colored area represents the standard deviation of the linear fitting.

Next, we performed the sequence of the repeated resonant readout and 532 nm charge control pulses (Figure 3(a)). The 445 nm blue pulse resets the PbV center to the dark state, as shown in Figure 2. In contrast to the result in Figure 2(b), the fluorescence from the negatively charged state gradually recovers as the 532 nm irradiation time increases (Figure 3(b, c)). Interestingly, in Figure 3(d), the repump rate has a nonlinear behavior with a near-quadratic exponent of 1.81(0.26), suggesting that the repump mainly originates from a two-photon process. Aside from Sample I, we also perform the pulse sequence experiment shown in Figure 3(a) on a color center in Sample II (see Methods) and observe a similar nonlinear behavior. Among four randomly selected PbV centers from the two samples, three exhibit a similar nonlinear behavior. One emitter exhibits a linear dependence, which is likely due to spectral diffusion, especially for the higher laser powers, leading to the weakened quadratic dependence. The reduced repump rate observed under a higher energy laser may also support this interpretation (SI Figure S3).

**Figure 3:**
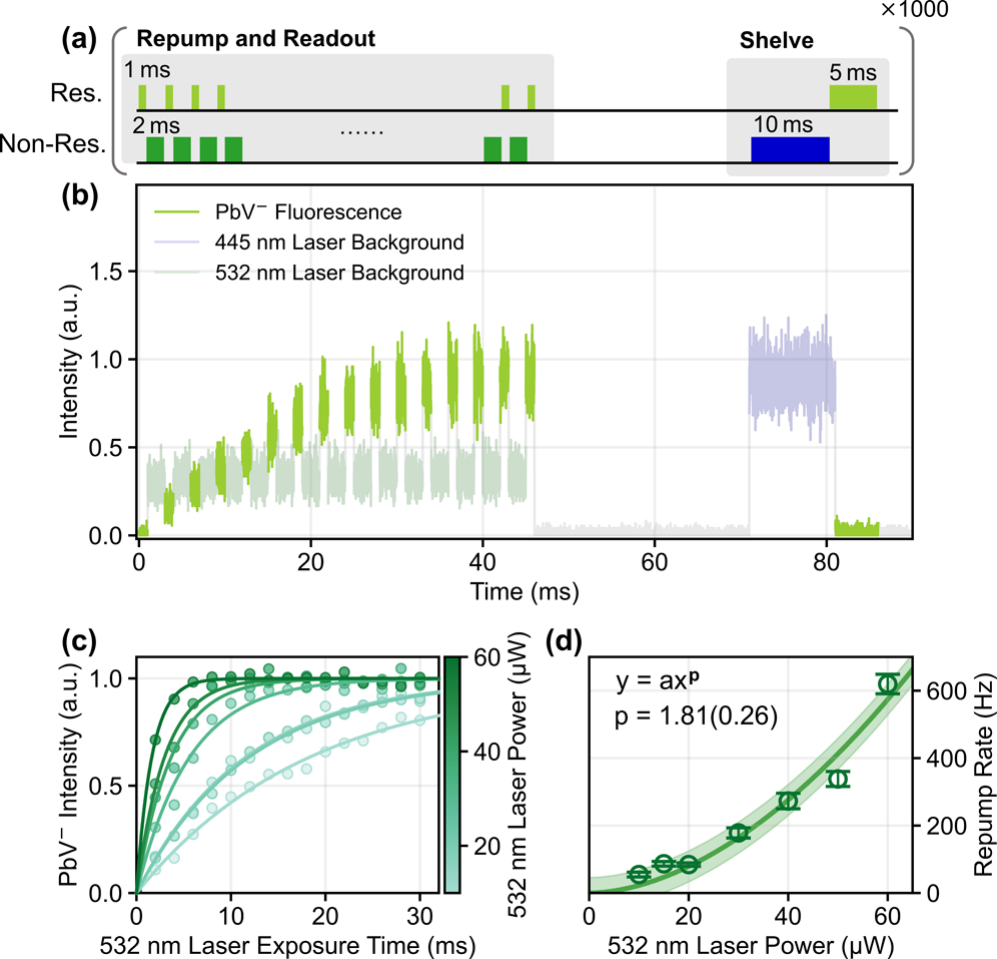
Time-resolved experiments of charge state initialization process. (a) Pulse sequence. (b) Fluorescence of a negatively charged PbV center, controlled by 532 nm laser irradiation. (resonant laser: 2 nW, 532 nm laser: 20 μW, 445 nm laser: 28.5 μW). (c) Transition curves using different 532 nm laser powers. (d) Transition rate as a function of the 532 nm laser power. Error bars are from the fitting error in panel (c), and the colored area represents the standard deviation of the power function fitting.

Based on the pulse experiments above, we find that the fluorescence intensity of the PbV center can be freely controlled with the nonresonant lasers. This means that we can also manipulate the population in a certain charge state. Figure 4 shows the population analysis for a PbV by using the sequence in Figure 3 (a) (see SI Figure S4 for details). Figure 4(a, b) shows the distributions of the detected photons in 1 ms resonant readouts repeated 1000 times with each 532 nm irradiation time and power. With the 532 nm irradiation (50 μW, 22 ms), a large peak centered at ∼15 photon count appears, corresponding to the bright negatively charged state, while the low photon counts (<3) regime is attributed to the dark state (Figure 4(a)). In contrast, without the 532 nm pulses after shelving with the 445 nm laser, we obtain only low counts in the histogram (Figure 4(b)), indicating the formation of a pure dark state after the 445 nm laser irradiation. Figure 4(c) summarizes the population of the negatively charged state as a function of the 532 nm laser power and duration. Saturation behaviors are observed at high powers and long irradiation time domains, and the maximum population into the negatively charged state is ∼89%.

**Figure 4:**
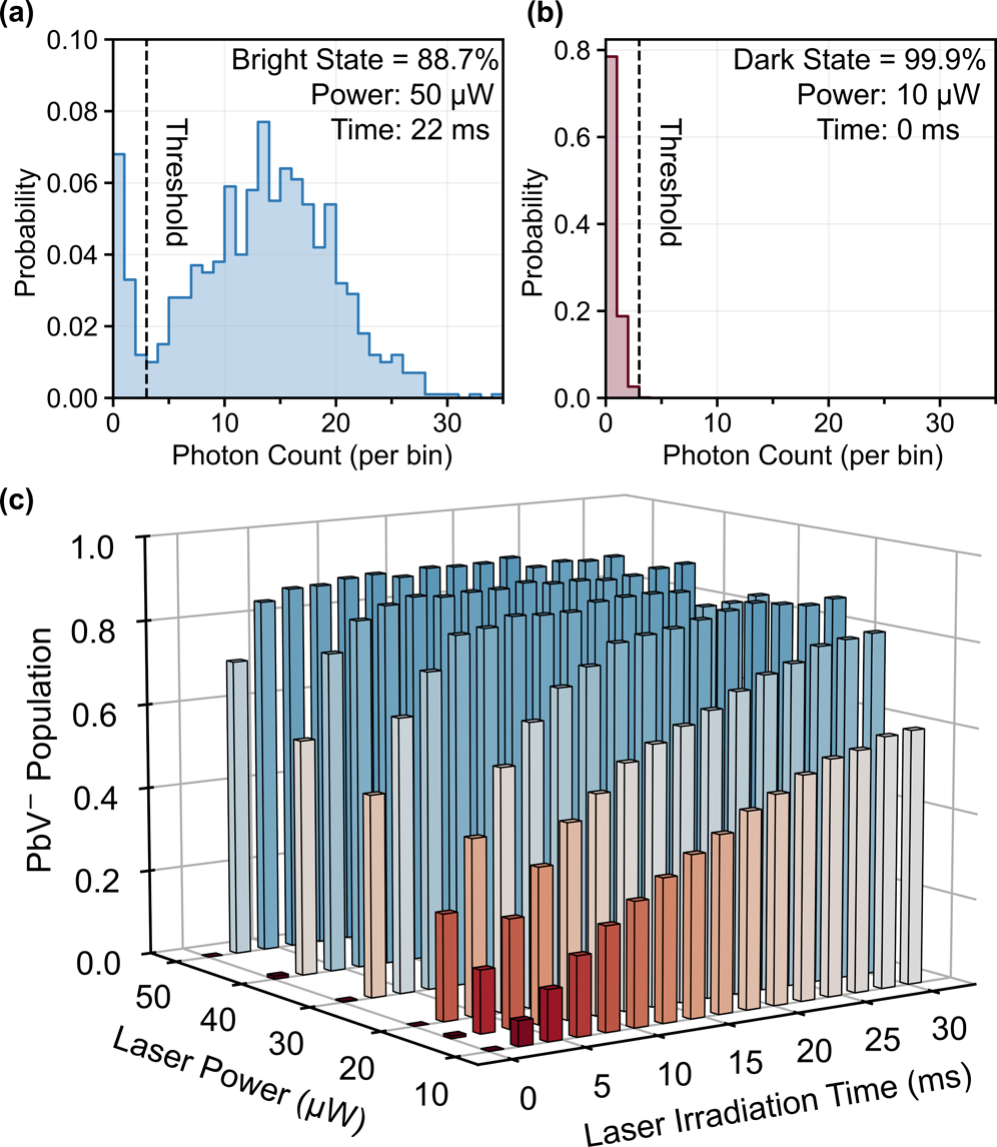
Manipulating the population of a PbV center. (a) Photon number distribution in a 1 ms charge state readout of a well-initialized case. (b) Photon number distribution after only 445 nm laser irradiation. (c) Population of the negatively charged state initialized at various 532 nm laser powers and times. The dashed lines in panels (a, b) are the threshold between the dark state and bright state that is set to three photons per bin.

Finally, we propose a model of the charge state transition of the PbV center under the nonresonant lasers. The calculated energy levels of the negatively charged and neutral states of the PbV center are demonstrated in Figure 5 (see SI for the computational method). First, we consider the shelving process caused by 445 nm laser irradiation (Figure 5(a)). The negatively charged PbV center has two possibilities for the photoionization: transition to the neutral state, or transition to the −2 negatively charged state. The transition to the neutral state by exciting an electron in the e_g_ level to the conduction band of diamond requires an optical energy of ∼2.6 eV,^38^ which is feasible with one 445 nm photon (2.79 eV). This agrees with the experimental observation in Figure 2(d). On the other hand, a higher energy of ∼3.5 eV is necessary for the transition to the −2 negatively charged state by capturing an electron from the valence band.^38^ Additionally, the recovery from the −2 charged state requires only one green photon (∼2.0 eV),^38^ again contradicting the observation in Figure 3(d). Thus, the dark state observed here is thought to be the neutral state.

**Figure 5:**
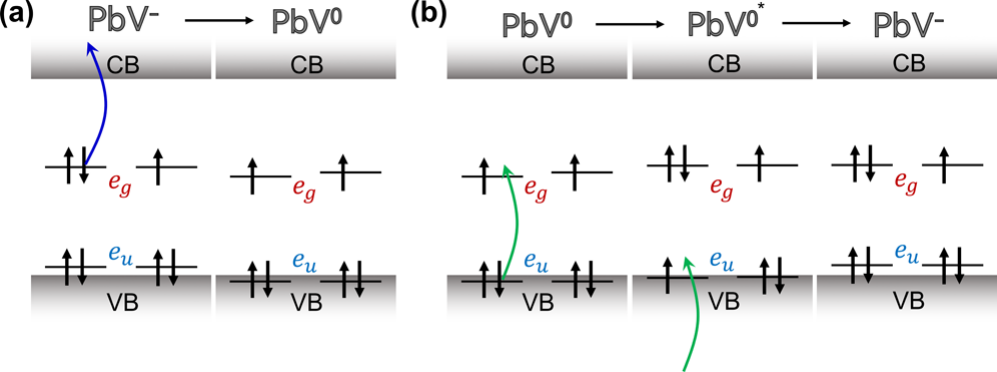
Proposed charge cycle process of the PbV center driven by (a) 445 and (b) 532 nm laser irradiation. VB and CB denote valence band and conduction band, respectively.

The recovery process experimentally occurs in a two-photon process under 532 nm laser irradiation (Figure 3(d)). This agrees with the previous calculation^38^ indicating that one 532 nm green photon (2.33 eV) does not have enough energy for the transition from the neutral to the −1 negatively charged state (∼2.9 eV). Accordingly, as shown in Figure 5(b), the first 532 nm photon is thought to excite the neutral PbV center into the excited state, creating a vacant state at the e_u_ level of the neutral state. Subsequently, the second 532 nm photon excites an electron from the valence band to occupy the vacant, consequently resulting in the charge state transition from the neutral state to the −1 negatively charged state. Thus, the neutral PbV initializes to the negatively charged state by two green photons.

It is worth noting that among the group-IV vacancy centers, the PbV center is only one emitter which can transition to the neutral state by visible blue laser irradiation according to the theoretical calculation.^38^ A lighter group-IV emitter has a charge transition level between the negatively charged state and neutral state closer to the valence band of diamond in the energy gap, and all levels of SiV, GeV, and SnV centers are below the midgap. Indeed, the visible blue laser experimentally repumps the SiV, GeV, and SnV center from the dark state into the negatively charged state.^29,31,39^ In contrast, the energy levels of the PbV center shift upward over the midgap, allowing the 445 nm laser to transition to the neutral state. Thus, the observed charge state transition is thought to be a unique process of the PbV center under 445 nm of irradiation.

Finally, to investigate potential fluorescence from the neutral state, we examine PL spectra under the nonresonant lasers (SI Figure S1). The blue laser leads to observation of only one small peak at 715 nm. This emission frequently appears in previous reports in Pb implanted diamonds.^6,40,41^ However, the emission wavelength is significantly different from predicted wavelengths of ∼560 nm for the neutral PbV center in theoretical calculations.^42,43^ Thus, we do not reach a conclusion about the origin of the observed 715 nm peak, and identification of the neutral charge state requires further investigations.

The charge cycle model we find here advances our understanding of the charge state of the PbV center in the diamond host material. Under 532 nm laser irradiation, we obtained a high population of up to 89% in the negatively charged state. This stable presence of the negatively charged state paves the way for the spin control of the PbV center, and establishes a solid foundation for spin single shot readout.^34^ It is worth noting that the charge dynamics is likely to be affected by the laser wavelength (SI Figure S3). Thus, the excitation wavelength dependence^27^ will be particularly interesting to fully understand the charge dynamics and stability of the PbV center. For the neutral state, we currently have no decisive evidence to determine whether the observed peak at 715 nm comes from the neutral PbV centers. Observation of the Stark effect of this emission line under external electric-fields could provide an insight into its atomic symmetry.^35^ It will also be important to investigate the predicted wavelength (∼560 nm). Since we do not see a peak at this wavelength under 445 nm laser irradiation, the addition of the resonant laser will enhance the excitation efficiency of this predicted line. Furthermore, the fabrication of a high-density PbV bulk sample could allow us to verify the existence of a spin-1 system in electron spin resonance measurements, as shown for the SiV center.^15^ Lastly, we mention that remote charge state conversion by capturing photocarriers occurs under 445 nm laser irradiation (SI Figure S5). Thus, we do not rule out the possibility that this process also plays a role in the charge state conversion observed above. However, direct laser irradiation should affect more efficiently, supported by the observation of a bright singularity spot for the charge state transition of NV, SiV, and PbV centers.^25,26,33,44^

We investigated the charge state control of the PbV center in diamond using multicolor nonresonant lasers. We first discovered that the 445 nm laser irradiation shelves the negatively charged PbV centers into a dark state, while the 532 nm irradiation repumps to the bright negatively charged state. Based on the detailed time-resolved experiment, we successfully established a charge state transition model for PbV centers when directly irradiated, which agrees with the ab initio theoretical results reported previously^38^ and current calculations. On the basis of this model, we propose that the dark state created by 445 nm laser direct irradiation is a neutral state, although the spectrum of the neutral charge state is still waiting to be discovered. Also, by combination of 445 and 532 nm lasers, we achieved the manipulation of negatively charged state PbV in the range of 0 to 89%. The saturation population of the negatively charged state also gives a crucial insight into charge state initialization, which is the most fundamental procedure for any spin control protocol on the IV-group defects in diamond. This indicates that the 532 nm laser can be used as a reliable source for high-fidelity charge state initialization.

## Supplementary Material



## Notes

Details of experimental and computational methods, PL spectra under 532 and 445 nm irradiation, resonant excitation quenching rate, laser power dependence of the repump rate, method for population analysis, and photocarrier induced transition to a dark state (PDF)
