# Supplementary material for: Optical Charge State Manipulation of Lead-Vacancy Centers in Diamond
Source: Nano Lett. 2025 Nov 13;25(47):16697–702. doi: 10.1021/acs.nanolett.5c04448 (PMC12670506; doi:10.1021/acs.nanolett.5c04448)
Supplement: Supplementary file 1 [file nl5c04448_si_001.pdf]

Supporting Information: Optical charge state manipulation of lead-vacancy centers in diamond

Yiyang Chen[1], Yoshiyuki Miyamoto[2], Eiki Ota[1], Ryotaro Abe[1], Takashi Taniguchi[3], Shinobu Onoda[4], Mutsuko Hatano[1], Takayuki Iwasaki[1,*]

[1]Department of Electrical and Electronic Engineering, School of Engineering, Institute of Science Tokyo, Meguro, 152-8552 Tokyo, Japan
[2]Advanced Power Electronics Research Center, National Instituteof Advanced Industrial Science and Technology, Tsukuba 305-8568 Ibaraki, Japan
[3]Research Center for Materials Nanoarchitectonics, National Institute for Materials Science, 305-0044 Tsukuba, Japan
[4]Takasaki Advanced Radiation Research Institute, National Institutes for Quantum Science and Technology, 1233 Watanuki, Takasaki, 370-1292 Gunma, Japan

[*]Email: iwasaki.t.c5b4@m.isct.ac.jp

1. Experimental and computational methods

For the fabrication of the PbV centers, Pb ions are implanted into two IIa-type (001) single-crystal diamond substrates with a fluence of 5×10^{8} cm^{-2} at an acceleration energy of 12 MeV [1]. To restore the lattice damage induced by the ion implantation and to form the PbV centers, high pressure and high temperature (HPHT) annealing is performed at 2300°C (Sample I) or 2200°C (Sample II) under 7.7 GPa for 20 min [1].

All optical experiments are conducted in a home-built confocal microscope. The samples are cooled down to about 6 K by a closed-cycle helium cryostat (s50, Montana Instrument). The laser light is guided to the sample by an apochromat ×50 objective lens (MPLAPON50× NA=0.95, OLYMPUS). Blue (06-MLD 445 nm, Cobolt) and green (06-DPL 532 nm, Cobolt) non-resonant lasers are used to control the charge state of the PbV centers. Optical pulses of these lasers are generated by an acousto-optic modulator (AOM, 532 nm, rise time 25 ns, Gooch & Housego) or direct modulation equipped with the laser (445 nm, rise time <2.5 ns). For resonant excitation, a dye tunable laser (Matisse 2 DS, Sirah Lasertechnik) and a tunable diode laser (DL-SHG pro, Toptica) are employed. A wavelength meter (WS8-30, HighFinesse) with a resolution of 1 MHz is used to monitor the wavelength and stabilize the tunable lasers through PID control. To stabilize the power of the dye laser into a range of 2 - 20 nW, the noise eater (NEL01A/M, Thorlabs) is used throughout the experiment. The resonant laser is gated using another AOM (532 nm, rise time 25 ns, Gooch & Housego). Phonon-side band (PSB) is detected through an optical filter for the resonant excitation. To control the timing and duration of the laser pulses, an arbitrary waveform generator (Pulse Streamer, Swabian Instruments) is employed. For all time-resolved experiments, we count the electrical pulse signals from an avalanche photo-diode (SPCM-AQRH-14, Excelitas) using a high-resolution fast counter (Time Tagger Ultra, Swabian Instruments). The confocal mapping and PLE scan are recorded by the Qudi framework [2].

The first-principles calculations are performed within the density functional theory. The plane-

wave basis set with cutoff energy of 64 Ry is used to express valence orbitals. Norm-conserving pseudopotentials [3] are used to express electron ion interactions. The pseudopotentials for Pb are constructed to treat Pb 5d orbitals as valence orbitals. The local density approximation using the functional form [4] is employed to express the exchange-correlation potentials. A supercell of 3×3×3 cubic diamond (216 C atoms) is used and the geometry optimization under electronic ground state is carried out by using the total-energy and force formalism [5]. All calculations are performed within the spin-unpolarized approximation with use of fractional occupation numbers. It should be noted that state-of-the-art calculations have been already performed for group-IV vacancy centers which includes spin-orbit interaction as well as dynamical Jahn-Teller effect [6]. Although these calculations are trustable, it is difficult to apply them to understand current experimental results intuitively. Therefore, here, approximated calculations are performed just to extract energy level change depending on the charged state, and on the ground/excited state configurations of electrons within the D_{3d} symmetry, which satisfactory works to understand the experimental results in this study.

2. PL spectra

Figure S1 shows PL spectra under continuous excitation with the different non-resonant lasers. We observe the C and D peaks of the negatively charged PbV centers at around 550 nm and also a peak around 715 nm under 532 nm excitation. In contrast, only a small peak at 715 nm is seen under the 445 nm excitation with background. The 715 nm emission has been reported under 490.5 nm, 520 nm, and 532 nm lasers in a previous work [7], but it could not be detected under laser excitation below 450 nm. In our work, we observe this peak with 445 nm laser in the HPHT annealed sample. As discussed in the main text, we need further studies to clarify the origin of this emission.

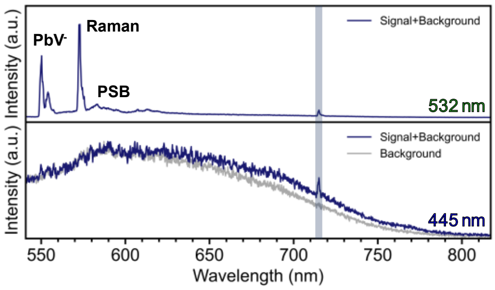

Figure S1. PL spectra. The excitation laser wavelength is (a) 532 nm and (b) 445 nm. A sharp peak around 572 nm is the first-order Raman scattering of diamond.

3. Resonant excitation quenching rate

Fluorescence quenching under resonant excitation is a well-reported phenomenon in the solid-state qubit [8–11]. We investigate the power dependence of the resonant excitation quenching rate (Fig. S2), showing a linear dependence on the resonant laser power with a slope of 0.08 Hz/nW. The resonant laser power used in the main text is set to 2 nW, which means that the resonant excitation quenching has little contribution to the charge state transition behavior induced by the non-resonant lasers in the main text.

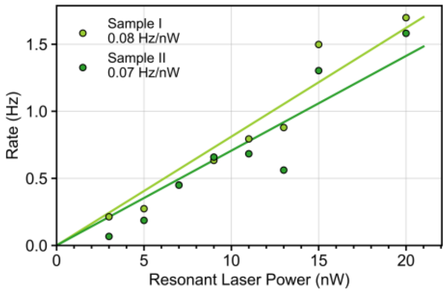

Figure S2. Power dependence of resonant excitation quenching rate for emitters in Sample I and Sample II.

4. Laser power dependence of the repump rate

We observe a non-linear behavior of the repump rate on the 532 nm laser power (Fig. 3(d)). We further investigate the laser power dependence on other PbV centers. Figure S3(a) shows the repump rate of four PbV centers including the curve in Fig. 3(d) (mentioned as PbV A). Three out of four emitters (PbV A, C, and D) demonstrate a nearly quadratic dependence on the 532 nm laser power, while PbV B shows a linear dependency. In addition, we perform the sequence to observe the repump rate using 515 nm laser. Surprisingly, the repump rate from the four emitters show different behaviors from those under 532 nm irradiation. The repump rate decreases overall and quadratic dependence is weakened using the 515 nm laser. For PbV B, the fitting largely deviates even from a linear dependence. We speculate that spectral diffusion may occur, especially under 515 nm irradiation: if the resonant readout becomes insufficient particularly for the higher laser powers, it leads to more significant decrease of the repump rate and weakened quadratic dependence. The average R^2 value of the fittings also supports this possibility: the data obtained using 532 nm laser yields an average R^2 of 0.97 while only 0.85 for 515 nm data. Unstable charge transition rates, which are likely caused by spectral diffusion, have been also observed for a SnV center [12]. A slightly shorter wavelength (higher energy) of the 515 nm laser would more efficiently ionize specific defects and thus might cause the unstable observation.

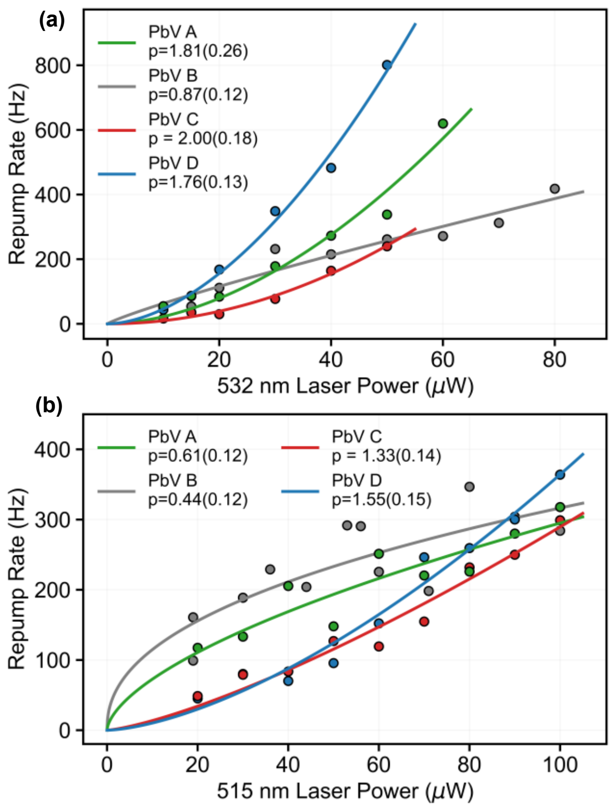

Figure S3. Repump rate of four different PbV centers under (a) 532 nm and (b) 515 nm laser irradiation. PbV A, B, and C are in Sample I and PbV D is in Sample II.

5. Population analysis

Figure S4 is the method for population analysis shown in the main text. The sequence consists of 16 resonant pulses (1 ms each) for the charge state readout, interleaved with 15 repump pulses of 532 nm laser. The sequence is repeated 1000 times, so that each resonant readout pulse contains 1000 bins of 1 ms duration. By unfolding these bins, we analyze the recorded photon number in each bin through frequency analysis, yielding the distributions presented in Figs. 4(a) and (b). Figure 4(c) is the summary of above analysis applied to 16 times resonant readouts at each 532 nm laser power and time.

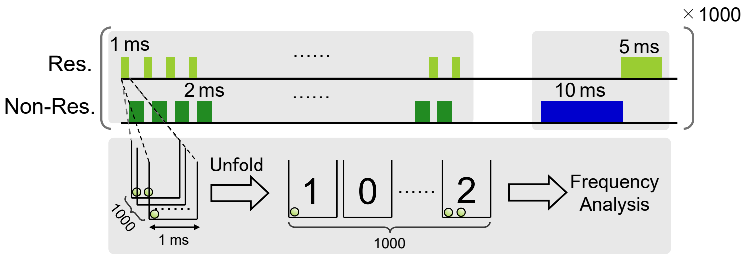

Figure S4. Method for population analysis.

6. Photo-carrier induced transition to a dark state

Figure S5(a) shows a scan image under resonant excitation. A narrow inhomogeneous distribution of the sample [1] enables us to observe five PbV centers at a fixed resonant wavelength. After irradiating a 445 nm laser pulse (1.2 mW, 5 ms) at a cross mark (×) in Fig. S5(a), we again perform a resonant scan at the same area while locking the tunable laser to the same wavelength. Within the circular region with a radius of 3.7 μm centered at the 445 nm laser irradiation spot, the PbV centers lose their fluorescence and turn dark (Fig. S5(b)). In contrast, the PbV centers outside the circle remain bright. This phenomenon is thought to be caused by the photo-carrier generation by the 445 nm pulse and the capture of the carriers by the PbV centers.

We further investigate laser power dependence of the photocarrier diffusion behavior at different distances (Fig. S5(c)). After a 445 nm laser pulse is applied at a location where neither PbV nor NV centers are present, a distance *D* away from the target PbV center, a resonant confocal scan is performed on the target PbV center to check the charge state: when a charge state conversion event is observed, the 532 nm laser is applied for repumping. But, without the charge conversion event, the focus point returns to the 445 nm laser irradiation point for another 445 nm laser pulse irradiation. Figure 3(c) shows the ionization probability by capturing the photo-carrier. Each data point is from 10 to 20 attempts of the remote charge state conversion. At a distance of 7 μm, the PbV center remains bright for the laser powers used here, except only one data in total 40 attempts. On the contrary, at a shorter distance of 3 μm, the charge state conversion occurrence saturates to 100% at an irradiation energy of ~2 μJ of the 445 nm laser. Although the sample formed at 2300°C suppresses the defect formation that can be excited by the 532 nm laser [1], we find here that there should be defects with deeper energy levels sensitive to the 445 nm laser. Interestingly, less error for the fitting of the ionization rate is observed under 445 nm irradiation (Fig. 2(d), R^2=0.99) compared to the 515 nm irradiation, suggesting that spectral diffusion is likely suppressed by 445 nm laser. Such behavior has been also reported on SiV and SnV centers using 445 nm laser [8,10].

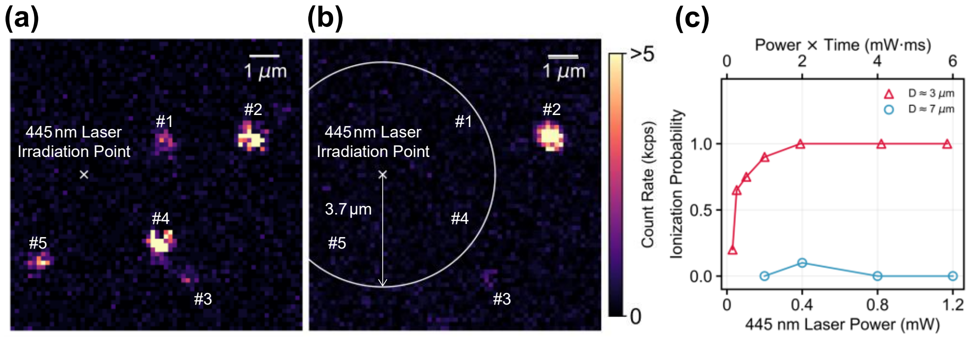

Figure S5. Photo-carrier assisted dark state transition under 445 nm irradiation. (a) Resonant scan image at a fixed laser wavelength. (b) Resonant scan image at the same laser wavelength as panel (a) after applying a 445 nm laser pulse at a cross mark. The contrast of the images is modified to enhance the visibility. (c) Probability of the charge state conversion of PbV centers by carrier diffusion induced by a 445 nm laser pulse.

Reference

[1] R. Abe, Y. Chen, P. Wang, T. Taniguchi, M. Miyakawa, S. Onoda, M. Hatano, and T. Iwasaki, Narrow Inhomogeneous Distribution and Charge State Stabilization of Lead-Vacancy Centers in Diamond, Adv. Funct. Mater. e12412 (2025).

[2] J. M. Binder et al., Qudi: A modular python suite for experiment control and data processing, SoftwareX **6**, 85 (2017).

[3] N. Troullier and J. L. Martins, Efficient pseudopotentials for plane-wave calculations, Phys. Rev. B **43**, 1993 (1991).

[4] J. P. Perdew and A. Zunger, Self-interaction correction to density-functional approximations for many-electron systems, Phys. Rev. B **23**, 5048 (1981).

[5] J. Ihm, A. Zunger, and M. L. Cohen, Momentum-space formalism for the total energy of solids, Journal of Physics C: Solid State Physics **12**, 4409 (1979).

[6] G. Thiering and A. Gali, Ab Initio Magneto-Optical Spectrum of Group-IV Vacancy Color Centers in Diamond, Phys. Rev. X **8**, 021063 (2018).

[7] S. Ditalia Tchernij et al., Spectral features of Pb-related color centers in diamond – a systematic photoluminescence characterization, New J. Phys. **23**, 063032 (2021).

[8] J. A. Zuber, M. Li, Marcel. li Grimau Puigibert, J. Happacher, P. Reiser, B. J. Shields, and P. Maletinsky, Shallow Silicon Vacancy Centers with Lifetime-Limited Optical Linewidths in Diamond Nanostructures, Nano Lett. **23**, 10901 (2023).

[9] D. Chen, Z. Mu, Y. Zhou, J. E. Fröch, A. Rasmit, C. Diederichs, N. Zheludev, I. Aharonovich, and W. Gao, Optical Gating of Resonance Fluorescence from a Single Germanium Vacancy Color Center in Diamond, Phys. Rev. Lett. **123**, 033602 (2019).

[10] J. Görlitz et al., Coherence of a charge stabilised tin-vacancy spin in diamond, Npj Quantum Inf **8**, 45 (2022).

[11] P. Wang et al., Transform-Limited Photon Emission from a Lead-Vacancy Center in Diamond above 10 K, Phys. Rev. Lett. **132**, 073601 (2024).

[12] K. Ikeda, Y. Chen, P. Wang, Y. Miyamoto, T. Taniguchi, S. Onoda, M. Hatano, and T. Iwasaki, Charge State Transition of Spectrally Stabilized Tin-Vacancy Centers in Diamond, ACS Photonics **12**, 2972 (2025).
